# Clinical indicators for recurrent cardiovascular events in acute coronary syndrome patients treated with statins under routine practice in Thailand: an observational study

**DOI:** 10.1186/s12872-015-0052-y

**Published:** 2015-06-16

**Authors:** Dujrudee Chinwong, Jayanton Patumanond, Surarong Chinwong, Khanchai Siriwattana, Siriluck Gunaparn, John Joseph Hall, Arintaya Phrommintikul

**Affiliations:** Department of Pharmaceutical Care, Faculty of Pharmacy, Chiang Mai University, Chiang Mai, Thailand; Clinical Epidemiology Program, Faculty of Medicine, Chiang Mai University, Chiang Mai, Thailand; Center of Excellence in Applied Epidemiology, Faculty of Medicine, Thammasat University, Pathum Thani, Thailand; Division of Medicine, Nakornping Hospital, Chiang Mai, Thailand; Department of Internal Medicine, Faculty of Medicine, Chiang Mai University, Chiang Mai, Thailand 50200; Centre for Clinical Epidemiology and Biostatistics, School of Medicine and Public Health, Faculty of Health, University of Newcastle, Callaghan, NSW Australia

**Keywords:** Subsequent cardiovascular events, LDL-C < 70 mg/dL, LDL-C goal, Multiple recurrent cardiovascular events, Acute coronary syndrome, eGFR, Revascularization

## Abstract

**Background:**

Acute coronary syndrome (ACS) patients are at very high cardiovascular risk and tend to have recurrent cardiovascular events. The clinical indicators for subsequent cardiovascular events are limited and need further investigation. This study aimed to explore clinical indicators that were associated with recurrent cardiovascular events following index hospitalization.

**Methods:**

The data of patients hospitalized with ACS at a tertiary care hospital in northern Thailand between January 2009 and December 2012 were retrospectively reviewed from medical charts and the electronic hospital database. The patients were classified into three groups based on the frequency of recurrent cardiovascular events (nonfatal ACS, nonfatal stroke, or all-cause death) they suffered: no recurrent events (0), single recurrent event (1), and multiple recurrent events (≥2). Ordinal logistic regression was performed to explore the clinical indicators for recurrent cardiovascular events.

**Results:**

A total of 405 patients were included; 60 % were male; the average age was 64.9 ± 11.5 years; 40 % underwent coronary revascularization during admission. Overall, 359 (88.6 %) had no recurrent events, 36 (8.9 %) had a single recurrent event, and 10 (2.5 %) had multiple recurrent events. The significant clinical indicators associated with recurrent cardiovascular events were achieving an LDL-C goal of < 70 mg/dL (Adjusted OR = 0.43; 95 % CI = 0.27–0.69, p-value < 0.001), undergoing revascularization during admission (Adjusted OR = 0.44; 95 % CI = 0.24–0.81, *p*-value = 0.009), being male (Adjusted OR = 1.85; 95 % CI = 1.29–2.66, *p*-value = 0.001), and decrease estimated glomerular filtration rate (Adjusted OR = 2.46; 95 % CI = 2.21–2.75, *p*-value < 0.001).

**Conclusion:**

The routine clinical practice indicators assessed in ACS patients that were associated with recurrent cardiovascular events were that achieving the LDL-C goal and revascularization are protective factors, while being male and having decreased estimated glomerular filtration rate are risk factors for recurrent cardiovascular events. These clinical indicators should be used for routinely monitoring patients to prevent recurrent cardiovascular events in ACS patients.

## Background

Acute coronary syndrome (ACS) is one of the clinical manifestations of cardiovascular diseases considered to be life threatening [1]. Comparing with the Global Registry of Acute Coronary Events (GRACE) [2] that showed an in-hospital mortality rate of 4.6 %; the in-hospital death rate was higher in the first [3] and second [4] Thai registries of ACS patients. Both are multi-center, prospective, nation-wide registries that collect relevant information in Thailand. The first Thai Acute Coronary Syndrome (TACS) registry [3] conducted between 2002 and 2004 in 17 provinces showed an in-hospital mortality rate of 12.6 %. Later, between 2007 and 2008, the second registry (the Thai Registry of Acute Coronary Syndrome, TRACS) was conducted in 39 provinces; it showed a reduced in-hospital morality of 4.8 %, but the mortality rates at 6-months and 1-year were still high (14.1 % and 17.7 %, respectively) [4].

Patients with established cardiovascular disease such as ACS patients are at higher risk for recurrent cardiovascular events following the first event [5–7], with about 1 % (140/13,608) [6] to 9 % (380/4,162) [7] of ACS patients having subsequent cardiovascular events. The first event of the composite of cardiovascular events was widely used in efficacy analyses for the Randomized Controlled Trials (RCTs) [8, 9], but the subsequent events following the first event are generally not considered in a primary end point analysis. However, in routine clinical practice both the patients and physicians are concerned not only about the first event but also about subsequent events. ACS patients with different frequency of recurrent cardiovascular events following their index hospitalization may differ in their clinical indicators. Investigating recurrent events, rather than only the first event, can provide more evidence for physicians and patients on how best to monitor patients’ progress. Some predictors of subsequent cardiovascular events such as age, high serum creatinine, and low high-density lipoprotein cholesterol were reported in survivors of first hospitalized myocardial infarction [10].

There are limited data available about the clinical indicators for recurrent cardiovascular events in Thailand. This study aims to explore if any of the information that is collected as part of routine clinical practice is associated with recurrent cardiovascular events in patients with ACS in Thailand.

## Methods

### Setting and study population

The study setting was the Maharaj Nakorn Chiang Mai Hospital, which is part of Chiang Mai University, with 1,400 patient beds to serve 1,300,000 outpatients and 48,000 inpatients annually [11]. This tertiary teaching hospital provides services to patients from Chiang Mai province (a population of approximately 1,600,000) and from 17 other provinces in northern Thailand that refer patients with complicated conditions such as ACS for specialist treatment. The hospital provides services in every medical discipline through a number of centers including the Northern Thailand Heart Center, the Northern Neuroscience Center, the Trauma Center, the Cancer Treatment and Research Center, the Respiratory Research Center, and the Lung Health Center. The research protocol was reviewed and approved by the Research Ethics Committee, Faculty of Medicine, Chiang Mai University, prior to commencement of data collection for the study.

We included all patients diagnosed with ACS - including unstable angina (UA), non-ST segment elevation myocardial infarction (NSTEMI), and ST segment elevation myocardial infarction (STEMI) - aged 18 years and over, treated with statins, and were admitted to the hospital between January 2009 and December 2012. A diagnosis of ACS was based on an ICD-10 (International Classification of Diseases, 10^th^ Revision) code of I20 (angina pectoris) or I21 (acute myocardial infarction). We retrospectively reviewed and retrieved the information for the clinical indicators of interest and cardiovascular events of the included patients from medical charts and from the electronic hospital database.

### Clinical indicators of interest

Clinical indicators of interest based on routinely clinical practice were collected: demographic data, co-morbidities, atherosclerotic risk factors, current medications, and laboratory results including lipid profiles (total cholesterol, low-density lipoprotein (LDL-C), high-density lipoprotein (HDL-C), and triglycerides), alanine aminotransferase (ALT), fasting blood glucose, and serum creatinine. The degree of renal function of patients was classified according to the estimated glomerular filtration rate (eGFR) during admission with the use of CKD-EPI Creatinine 2009 Equation, which estimated eGFR from serum creatinine, age, sex, and race, into two groups: < 60 mL/min/1.73 m^2^ and ≥ 60 mL/min/1.73 m^2^ [12]. LDL-C goal attainment was determined at the first follow-up visit of patients which occurred between 2 weeks and 1 year from the admission date. LDL-C levels were categorized into one of three groups: LDL-C < 70 mg/dL, 70–99 mg/dL, and ≥ 100 mg/dL; LDL-C < 70 mg/dL (<1.8 mmol/L) was classified as achieving the LDL-C goal according to the guidelines [13]; LDL-C ≥ 100 mg/dL was used as the reference group in the analysis. Revascularization was defined as undergoing percutaneous coronary intervention (PCI) or coronary artery bypass surgery (CABG) during admission of patients.

### Recurrent cardiovascular events

**Fig. 1:**
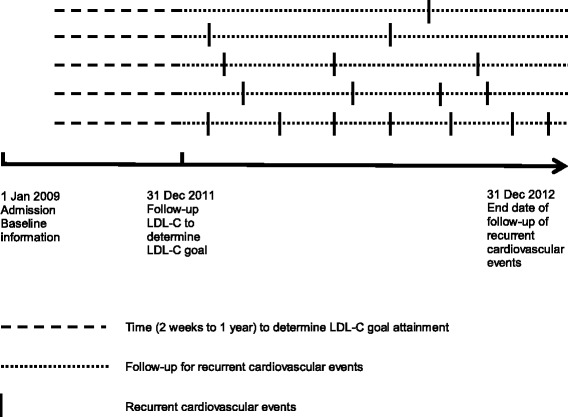
Index date, study period, and recurrent cardiovascular events

In our study, recurrent cardiovascular events were defined as nonfatal ACS (myocardial infarction (MI) or unstable angina), nonfatal stroke, or all-cause death following the index hospitalization. Patients were categorized into three groups based on the frequency of recurrent cardiovascular events: no recurrent event (0), single recurrent event (1), and multiple recurrent events (≥2, Fig. 1). For example, if a patient experienced only a nonfatal MI, this was classified as having a single recurrent event. If a patient had a nonfatal MI, and the same patient subsequently had a stroke, the patient was characterized as having multiple recurrent events. Using this method, all events were weighted equally (i.e. death and recurrent MI or stroke were weighted equally).

### Statistical analysis

Descriptive statistics were examined to describe variables with counts and percentages reporting for categorical variables, and means with standard deviations for continuous variables. We used nonparametric tests for trends across ordered groups to investigate differences across the three groups of patients. Due to the ordinal nature of the outcome variable (0, 1, ≥2 recurrent events), we used ordinal logistic regression [14, 15]. Univariable and multivariable ordinal logistic regression (clustered with stratum of ACS [UA, NSTEMI, STEMI] and adjusted with the length of follow-up time) were performed to explore the clinical indicators for recurrent cardiovascular events. The two-tailed test was used and p-value < 0.05 was considered statistically significant. All analyses were carried out using STATA software, version 12 (StataCorp LP, College Station, TX, USA).

## Results

A total of 1,089 medical records of patients diagnosed with ACS were reviewed. Due to the incompleteness of the essential data for analysis, lack of LDL-C level at baseline and follow-up, we excluded 684 patients’ records, resulting in 405 patients being included in the final analysis. We performed a comparison analysis between those patients excluded and included in the analysis and found that the two groups were not significantly different in their baseline characteristics; but the excluded patients were older than the included patients (67.2 ± 12.9 vs 64.9 ± 11.5; *p*-value = 0.003).

In our study, the median time of follow-up from index hospitalization to the last medical contact, or until 31 December 2012, was 810 days (Interquartile range [IQR]: 489–1093). For those with a single recurrent event (36 patients), the median time from index hospitalization to the first recurrent event was 278 days (IQR: 159–522). Of the 405 patients, 359 (88.6 %) patients did not experience any recurrent event; 36 (8.9 %) patients experienced a single recurrent event, and 10 (2.5 %) patients experienced ≥ 2 recurrent events. The three groups were similar in gender, age, health insurance status, smoking status, having dyslipidaemia, having a family history of premature atherosclerosis, having a previous history of chronic stable angina, stroke, and peripheral vascular disease, having a history of CABG and carotid intervention, and current medication use. They also were similar in most of the laboratory findings except for serum creatinine and eGFR. Characteristics that differed among groups were diagnosis at discharge, diabetes mellitus, hypertension, chronic kidney disease, previous histories of MI or UA, previous histories of percutaneous coronary intervention (PCI), undergoing PCI during admission, current medication with diabetic drugs and calcium channel blocker (CCB) (Table 1). Of those who had a recurrent cardiovascular event, nonfatal ACS was the most common; ten patients died; ten patients had multiple recurrent cardiovascular events; one patient had seven cardiovascular events (all nonfatal ACS) (Table 2).

**Table 1:** Baseline characteristics of acute coronary syndrome patients with no cardiovascular events, a single event, or multiple events (n = 405)

Characteristics	Recurrent cardiovascular events			*p*-value for trend
	**0**	**1**	**≥2**	
	**(n = 359)**	**(n = 36)**	**(n = 10)**	
Gender				
Male	215 (60.0)	24 (66.7)	6 (60.0)	0.600
Age, (year)	64.5 ± 11.5	68.1 ± 11.7	66.7 ± 11.6	0.128
Health insurance				
Universal coverage scheme	201 (56.0)	19 (52.8)	5(50.0)	0.630
Civil servant medical benefit scheme	139 (38.7)	16 (44.4)	4 (40.0)	
Social security scheme	15 (4.2)	1 (2.8)	1 (10.0)	
Self- pay	4 (1.1)	0 (0.0)	0 (0.0)	
Smoking				
Non smoker	208 (57.9)	23 (63.9)	8 (80.0)	0.204
Ex-smoker	67 (18.7)	6 (16.7)	0 (0.0)	
Current smoker	84 (23.4)	7 (19.4)	2 (20.0)	
Diagnosis at discharge				
Unstable angina	66 (18.4)	5 (13.9)	7 (70.0)	0.001
NSTEMI	90 (25.1)	16 (44.4)	2 (20.0)	
STEMI	203 (56.6)	15 (41.7)	1 (10.0)	
Atherosclerotic risk factors				
Diabetes mellitus	97 (27.0)	15 (41.7)	5 (50.0)	0.019
Hypertension	215 (59.9)	29 (80.6)	7 (70.0)	0.039
Chronic kidney disease	37 (10.3)	12 (33.3)	2 (20.0)	0.001
Dyslipidemia	141 (39.3)	17 (47.2)	6 (60.0)	0.119
Family history of premature atherosclerosis	7 (1.9)	0 (0.0)	0 (0.0)	0.369
Previous history of cardiovascular events				
Chronic stable angina	30 (8.4)	2 (5.6)	3 (30.0)	0.174
Myocardial infarction or unstable angina	71 (19.8)	11 (30.6)	5 (50.0)	0.008
Stroke (Ischemic)	22(6.1)	1 (2.8)	1 (10.0)	0.870
Peripheral vascular disease	1 (0.3)	0 (0.0)	0 (0.0)	0.736
Previous history of cardiovascular intervention				
PCI	19 (5.3)	4 (11.1)	3 (30.0)	0.002
CABG	17 (4.7)	0 (0.0)	3 (30.0)	0.071
Revascularization of peripheral vascular disease	1 (0.3)	0 (0.0)	0 (0.0)	0.736
Carotid intervention	2 (0.6)	0 (0.0)	0 (0.0)	0.633
Treatment during admission				
PCI	151 (42.1)	7 (19.4)	1 (10.0)	0.001
CABG	4 (1.1)	1 (2.8)	0 (0.0)	0.735
Thrombolytic indicated	43 (12.0)	6 (16.7)	1 (10.0)	0.690
Medications				
Lipid lowering drugs (non-statins)	9 (2.5)	1 (2.8)	1 (10.0)	0.271
Antiplatelet/Anticoagulant drugs	350 (97.5)	35 (97.2)	10 (100.0)	0.766
Beta-blockers	296 (82.5)	31 (86.1)	9 (90.0)	0.414
ACEI/ARB	235 (65.5)	18 (50.0)	5 (50.0)	0.054
CCB	71 (19.8)	7 (19.4)	7 (70.0)	0.006
Diuretics	100 (27.9)	14 (38.9)	1 (10.0)	0.979
Diabetic drugs	53 (14.8)	8 (22.2)	4 (40.0)	0.021
Baseline laboratory results				
Serum creatinine (mg/dL)	1.4 ± 1.8	1.8 ± 1.1	1.3 ± 0.4	0.003
eGFR (mL/min/1.73 m^2^)	62.7 ± 25.7	46.3 ± 23.0	58.5 ± 21.8	0.004
ALT (U/L)	35.4 ± 45.5	41.5 ± 88.8	26.6 ± 10.0	0.142
Fasting blood glucose (mg/dL)^a^	135.1 ± 75.3	134.1 ± 47.7	164.4 ± 70.5	0.230
Total cholesterol (mg/dL)	181.1 ± 50.4	185.4 ± 43.5	169.8 ± 46.8	0.757
Triglyceride (mg/dL)^b^	137.0 ± 81.2	158.7 ± 89.5	196.4 ± 153.9	0.086
High density lipoprotein (mg/dL)^c^	40.4 ± 11.7	38.0 ± 7.5	34.8 ± 9.7	0.168
Low density lipoprotein (mg/dL)	112.6 ± 41.9	114.0 ± 37.9	92.3 ± 29.6	0.300
Median follow-up time (day)^d^	808 (490–1,073)	782 (306–1,146)	1,088 (674–1,239)	0.609

**Abbreviations:** LDL-C, low-density lipoprotein cholesterol; mg/dL, milligrams per deciliter; NSTEMI, non –ST segment elevation myocardial infarction; STEMI, ST segment elevation myocardial infarction; PCI, percutaneous coronary intervention; CABG, coronary artery bypass surgery; ACEI/ARB, angiotensin-converting enzyme inhibitors/angiotensin II receptor blockers; CCB, calcium channel blocker; eGFR, estimated glomerular filtration rate; ALT, alanine aminotransferase; U/L, units/liter

**Notes:** Numbers are n (%) or mean ± standard deviation (SD) or median (Interquartile range); the data were missing for some variables, ^a^fasting blood glucose, n = 343, 35, 9; ^b^triglyceride, n = 335, 32, 5; ^c^ high density lipoprotein, n = 335, 32, 5; ^d^ time form index hospitalization to last medical contact

**Table 2:** Summary of recurrent cardiovascular events

Recurrent events (n = 46)	Patients with event
Single recurrent event (n = 36)	
MI	26
Stroke	0
Cardiovascular death	5
Non-cardiovascular death	5
Multiple recurrent events (n = 10)	
Two recurrent events	6
MI, stroke	1
Stroke, MI	1
MI, MI	4
Three recurrent events (all nonfatal ACS)	2
Four recurrent events (all nonfatal ACS)	1
Seven recurrent events (all nonfatal ACS)	1

**Abbreviations:** MI, myocardial infarction; ACS, acute coronary syndrome

The univariable ordinal logistic regression showed that the significant clinical indicators associated with recurrent cardiovascular outcomes were achieving LDL-C goal of < 70 mg/dL, revascularization, eGFR <60 mL/min/1.73 m^2^, increased age, hypertension, use of angiotensin-converting enzyme inhibitors (ACEI/ARB) (Table 3). With multivariable ordinal logistic regression, four clinical factors (2 protective factors and 2 risk factors) associated with recurrent cardiovascular events were achieving LDL-C goal of < 70 mg/dL (Adjusted OR = 0.43; 95 % CI = 0.27–0.69, *p*-value < 0.001), undergoing revascularization during admission (Adjusted OR = 0.44; 95 % CI = 0.24–0.81, *p*-value = 0.009), being male (Adjusted OR = 1.85; 95 % CI = 1.29–2.66, *p*-value = 0.001), and eGFR < 60 mL/min/1.73 m^2^ (Adjusted OR = 2.46; 95 % CI = 2.21–2.75, *p*-value < 0.001) (Table 3). In our study, there were five non-cardiovascular deaths; nevertheless, the results of clinical indicators on recurrent cardiovascular events were consistent when using cardiovascular death instead of all-cause death. In addition, ACEI/ARB was found to be a protective factor for recurrent events (the data not shown).

**Table 3:** Univariable and multivariable analysis of clinical indicators for recurrent cardiovascular events (n = 405)

Clinical indicators	OR (95 % CI)	*p*-value	Multivariable OR (95 % CI)	*p*-value
LDL-C goal attainment				
LDL-C ≥ 100 mg/dL	1.00		1.00	
LDL-C 70–99 mg/dL	0.75 (0.36–1.58)	0.448	0.67 (0.35–1.30)	0.240
LDL-C < 70 mg/dL	0.55 (0.33–0.91)	0.019	0.43 (0.27–0.69)	<0.001
Revascularization	0.32 (0.17–0.63)	0.001	0.44 (0.24–0.81)	0.009
eGFR < 60 mL/min/1.73 m^2^	3.24 (2.74–3.82)	<0.001	2.46 (2.21–2.75)	<0.001
Male gender	1.25 (0.79–1.96)	0.337	1.85 (1.29–2.66)	0.001
Age (year)	1.03 (1.01–1.04)	<0.001	1.00 (0.99–1.03)	0.258
Hypertension	2.39 (1.20–4.73)	0.013	1.66 (0.70–3.95)	0.249
ACEI/ARB	0.53 (0.35–0.81)	0.003	0.72 (0.49–1.06)	0.101
Diabetes mellitus	2.09 (0.67–6.50)	0.202	1.56 (0.52–4.73)	0.428
Follow-up time (day)^a^	1.00 (1.00–1.00)	0.908	1.00 (1.00–1.00)	0.890

**Abbreviations:** OR, odds ratio; CI, confidence interval; LDL-C, low-density lipoprotein cholesterol; mg/dL, milligrams per deciliter; eGFR, estimated glomerular filtration rate; ACEI/ARB, angiotensin-converting enzyme inhibitors/angiotensin II receptor blockers

**Note:** ^a^time from index hospitalization to the last medical contact

## Discussion

In our study, multiple recurrent cardiovascular events occurred in 2.5 % of ACS patients, which are in line with previous studies that 1–9 % of patients had multiple recurrent cardiovascular events. Our study to investigate the clinical factors that were associated with recurrent cardiovascular events identified two protective factors – achieving LDL-C goal of less than 70 mg/dL, and undergoing revascularization (either PCI or CABG) during admission. The study also found two risk factors for further events – male gender and decreased eGFR.

### Achieving LDL-C goal of less than 70 mg/dL

Our finding shows that patients with ACS who achieve the LDL-C goal of less than 70 mg/dL have fewer recurrent cardiovascular events compared to those not achieving goal. To our knowledge, there is no other study that investigates the association between LDL-C goal achievement and recurrent cardiovascular events. However, some studies [16–18], including our former study [19], demonstrated that lowering LDL-C to less than 70 mg/dL resulted in reducing the incidence of cardiovascular events. Our previous study revealed that ACS patients treated with statins who achieved an LDL-C goal of <70 mg/dL had significantly fewer composite cardiovascular outcomes [19]. Similarly, the results from the two post-hoc analyses from the PROVE IT-TIMI 22 RTC (Pravastatin or Atorvastatin Evaluation and Infection Therapy–Thrombolysis In Myocardial Infarction 22) [17, 18] showed that ACS patients with the lower LDL-C values (≤40 mg/dL and >40 to 60 mg/dL groups) had a reduction in cardiac events (death, MI, stroke, recurrent ischemia, revascularization) when compared with the reference group (>80 to 100 mg/dL) [17]. The same study found that elderly patients with ACS who attained LDL-C levels < 70 mg/dL had a 40 % relative lower risk of acute cardiac clinical events of death, MI, or UA requiring rehospitalisation [18].

Further, a recently released result of a RCT study, the IMProved Reduction of Outcomes: Vytorin Efficacy International Trial (IMPROVE-IT) [20–22], conducted over 9 years on 18,144 patients with post-ACS from 39 countries, showed that an LDL-C less than 60 mg/dL is associated with a reduction in cardiovascular events. The primary end point of that study was a composite of cardiovascular death, MI, unstable angina requiring rehospitalisation, coronary revascularization, or stroke. The primary endpoint in the ezetimibe plus simvastatin group, with LDL-C of about 53 mg/dL after 1 year of follow-up, was decreased by 6.4 % over 7 years when compared with the simvastatin (40 mg) only group, with LDL-C of about 69 mg/dL (p = 0.016).

Many guidelines, such as the ESC/EAS Guidelines for the management of dyslipidemias [13], and the 2014 National Lipid Association [23], recommend an LDL-C goal of less than 70 mg/dL as a target for therapy in ACS patients. Recently, some guidelines – 2013 ACC/AHA on cholesterol management [24] and the NICE guidelines on lipid modification [25] – does not recommend the LDL-C goal because they found no evidence from RCTs studies to confirm an association between treating to the LDL-C target and cardiovascular events or mortality. As a result, the treating to target approach has been debatable in lipid management for some physicians. Our finding supports that treating to an LDL-C target of less than 70 mg/dL is beneficial because patients who do not achieve this goal are more likely to have subsequent cardiovascular events. This suggests that physicians should discuss with patients the importance of getting their LDL-C goal below 70 mg/dL to reduce their risk of further cardiovascular events.

### Revascularization

Our findings show that undergoing revascularization, either with PCI or CABG, is associated with fewer subsequent cardiovascular events. To our knowledge, no studies have been conducted to assess the impact of revascularization on recurrent cardiovascular events. Nevertheless, previous studies [26–31] showed improvement in the clinical outcomes of ACS patients who underwent revascularization procedures during hospitalization. For example, a study conducted by Held et al. revealed that revascularization within 14 days of hospital admission for ACS was associated with a significant 30 % reduction in 1-year mortality [26]. The results of the Canadian ACS Registry showed that in-hospital revascularization was associated with better 1-year survival only among patients with high-risk non–ST- elevation acute coronary syndrome [27]. Vanasse et al. demonstrated that patients with myocardial infarction who underwent revascularization had a better 2-year cardiovascular survival rate compared to patients without revascularization, regardless of pharmacological treatments [31].

It has to be noted that there was higher prevalence of revascularization in this study than in the two registries of ACS patients in Thailand, possibly because this study was conducted in a University hospital where all patients were managed by cardiologists, while the two Thai ACS registries reported on a variety of hospitals with different capabilities [3, 4]. Also, the proportion of ACS patients that underwent revascularization is higher than that in a study in Sri Lanka where no patients presenting with STEMI underwent PCI or CABG [32].

### Male gender

The association between gender and mortality among the patients with cardiovascular disease is inconclusive [33–37]. In our study more males died than females; of ten deaths, six were males. However, this total is too low for generalizations. We also found that males were more likely to have recurrent cardiovascular events; this is consistent with a study by Wilson et al. that being male was a significant predictor of recurrent cardiovascular events [34]. However, Movahed et al. found a higher mortality rate among women undergoing percutaneous coronary intervention in comparison to men [35]. Singh et al. reported no significant differences between men and women patients after PCI in short-term (30-day mortality) or long-term mortality, after accounting for risk factors [36]. Similarly, a study by D’Ascenzo et al. found similar long-term major adverse cardiac events between the female and male patients undergoing PCI [33].

### Decreased eGFR

Elevated serum creatinine and decreased eGFR suggest impaired renal function, with eGFR being a more reliable indicator [1]. Studies showed that increased serum creatinine or decreased eGFR was associated with major adverse cardiac events [38–46]. Our finding adds to that knowledge i.e. renal dysfunction, based on eGFR < 60 mL/min/1.73 m^2^, is associated with recurrent cardiovascular events. This observation is in line with previous studies that renal dysfunction was found to predict the likelihood of recurrent cardiovascular disease [10, 47].

### Limitations

Due to the limitations of this study, the results should be interpreted with caution. The first limitation is related to the nature of retrospective study design, in that residual and/or unknown confounding factors could exit, and some data were unavailable. For example, the time from hospital admission of the ACS patients to the assessment of LDL-C goal attainment varied from 2 weeks to one year, depending on the availability of the patients’ lipid profiles on the first follow-up visit. As per the ESC/EAS Guidelines for the management of dyslipidaemias [13], patients’ lipids should be tested 4–12 weeks after starting lipid-lowering treatment. In our study, few patients (25, 6.2 %) had an LDL-C measurement before 4 weeks. Second, all patients included in the study had a very high cardiovascular risk (ACS patients), so the findings may not apply to patients with less severe disease. In addition, all patients were treated by cardiologists at a University hospital where the level of care exceeds that in lower level hospitals. Third, the number of patients with the occurrence of recurrent events was also very low (36 patients or 8.9 % with single recurrent event, and 10 patients or 2.5 % with multiple recurrent events), so that larger scale studies are required before the relationships found here can be generalized. Fourth, although some biomarkers have been shown to be independent prognostic markers for morbidity and mortality in ACS patients, e.g., B-type natriuretic peptide and high-sensitivity C-reactive protein [48, 49], these biomarkers have not been routinely measured in clinical practice in our setting. Biomarkers therefore were not included as potential clinical indicators for recurrent events in our study.

However, only a few studies have assessed the relationship between LDL-C goal attainment and cardiovascular events, and even fewer looked at subsequent cardiovascular events in a real-world setting; our study provides information about the factors associated with recurrent cardiovascular events in ACS patients in real world practice based on information collected as part of routine clinical practice. Our findings will be of use to physicians to identify ACS patients at higher risk of recurrent cardiovascular events who should be intensively followed up to prevent subsequent cardiac events, namely those ACS patients who do not achieve the LDL-C goal of < 70 mg/dL, did not undergoing revascularization, are male, and have decreased eGFR.

## Conclusion

In conclusion, this study of routine clinical practice in ACS patients found that achieving an LDL-C goal of less than 70 mg/dL and undergoing revascularization are protective factors, whereas male gender and an eGFR less than 60 mL/min/1.73 m^2^are risk factors for recurrent cardiovascular events. These clinical indicators should be used for routine-monitoring of patients to prevent recurrent cardiovascular events in ACS patients.

## Abbreviations

ACS: Acute coronary syndrome
GRACE: Global Registry of Acute Coronary Events
TACS: Thai Acute Coronary Syndrome
TRACS: Thai Registry of Acute Coronary Syndrome
ICD-10: International Classification of Diseases, 10^th^ Revision
LDL-C: Low-density lipoprotein cholesterol
HDL-C: High-density lipoprotein cholesterol
IMPROVE-IT: IMProved Reduction of Outcomes: Vytorin Efficacy International Trial
UA: Unstable angina
NSTEMI: Non-ST segment elevation myocardial infarction
STEMI: ST segment elevation myocardial infarction
MI: Myocardial infarction
PCI: Percutaneous coronary intervention
CABG: Coronary artery bypass surgery
ACEI/ARB: Angiotensin-converting enzyme inhibitors/angiotensin II receptor blockers
CCB: Calcium channel blocker
